# Deleterious germline *CARD11* gain-of-function variants alter human B-cell and CD4^+^ T-cell differentiation and function

**DOI:** 10.1093/cei/uxag034

**Published:** 2026-06-12

**Authors:** Tina Nguyen, Melissa A Kallarakal, Jackie L Ludgate, Ian M Morison, Helen C Su, Swadinhya Arjunaraja, Andrew L Snow, Cindy S Ma, Stuart G Tangye

**Affiliations:** Garvan Institute of Medical Research, Darlinghurst, NSW, Australia; School of Clinical Medicine, Faculty of Medicine and Health, UNSW Sydney, Kensington, NSW,Australia; Department of Pharmacology & Molecular Therapeutics, Uniformed Services University of the Health Sciences, Bethesda, MD, USA; Department of Haematology, Dunedin School of Medicine, University of Otago, Dunedin, New Zealand; Department of Haematology, Dunedin School of Medicine, University of Otago, Dunedin, New Zealand; Awanui Labs, Dunedin, New Zealand; Human Immunological Diseases Section, Laboratory of Clinical Immunology and Microbiology, Intramural Research Program, NIAID, NIH, Bethesda, MD, USA; Department of Pharmacology & Molecular Therapeutics, Uniformed Services University of the Health Sciences, Bethesda, MD, USA; Department of Pharmacology & Molecular Therapeutics, Uniformed Services University of the Health Sciences, Bethesda, MD, USA; Garvan Institute of Medical Research, Darlinghurst, NSW, Australia; School of Clinical Medicine, Faculty of Medicine and Health, UNSW Sydney, Kensington, NSW,Australia; Garvan Institute of Medical Research, Darlinghurst, NSW, Australia; School of Clinical Medicine, Faculty of Medicine and Health, UNSW Sydney, Kensington, NSW,Australia

**Keywords:** CARD11 gain-of function, hypogammaglobulinemia, immunodeficiency, human B and CD4^+^ T cells

## Abstract

CARD11 is a scaffold protein expressed primarily in hematopoietic tissues, shaping key processes in B and T cells via regulation of Ag-linked signaling pathways, including NF-κB, mTOR, JNK, and AKT. Heterozygous, gain-of-function (GOF) variants in its encoding gene, CARD11, are implicated in a human disorder characterized by frequent upper respiratory infections, poor responses to polysaccharide vaccines, vulnerability to certain opportunistic viruses, and polyclonal B-cell expansion, likely predisposing these patients to lymphoma. Over the past decade, several studies have elucidated some of the B-cell functional defects that likely underlie the patients’ infectious phenotype. However, the potential contributions of CARD11 GOF variants to subpopulations of T cells have not been explored in detail. Therefore, our study sought to investigate the effect of increased CARD11 activity on the development, maturation, activation, differentiation, and effector function of adaptive lymphocytes from a cohort of five individuals harboring monoallelic CARD11 GOF variants through detailed ex vivo immunophenotyping and in vitro analyses. Our findings revealed intrinsic requirements for CARD11 in activation, differentiation, and effector function of human naïve B cells. Contrary to previous reports, intact CARD11 activity is also required for multiple aspects of CD4+ T-cell homeostasis alongside its notable role in the humoral immune response. Overall, these results shed light on mechanisms underlying disease pathogenesis due to not only CARD11 GOF variants but also LOF variants and reveal opportunities to consider targeted therapies in CARD11 GOF patients.

## Teaser

CARD11 is a scaffold protein expressed primarily in hematopoietic tissues, shaping key processes in B and T cells via regulation of Ag-linked signaling pathways including NF-κB, mTOR, JNK, and AKT. Heterozygous, gain-of-function (GOF) variants in its encoding gene, CARD11, are implicated in a human disorder characterized by frequent upper respiratory infections, poor responses to polysaccharide vaccines, vulnerability to certain opportunistic viruses, and polyclonal B-cell expansion, likely predisposing these patients to lymphoma. Our study sought to investigate the effect of increased CARD11 activity on the development, maturation, activation, differentiation and effector function of adaptive lymphocytes from a cohort of five individuals harboring monoallelic CARD11 GOF variants through detailed ex vivo immunophenotyping and in vitro analyses.

## Graphical Abstract

**Graphical Abstract:**
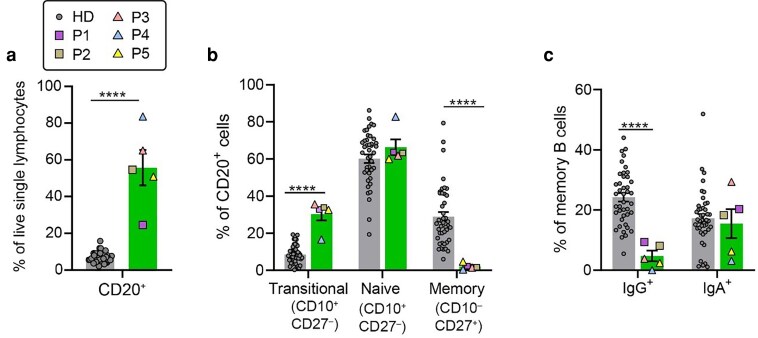

## Introduction

Caspase Recruitment Domain Family Member 11 (CARD11) is a ∼130 kDa scaffold protein belonging to the extensive family of membrane-associated guanylate kinases (MAGUKs), which facilitate assembly of protein complexes for intracellular signal transduction and neuronal synapse function [1–3]. In lymphocytes, CARD11 transmits signals from the T- and B-cell antigen (Ag) receptors (TCR, BCR) to regulate multiple intracellular pathways. Specifically, conformational changes in CARD11 downstream of Ag receptor engagement enables recruitment of BCL10 (B-cell lymphoma 10) and MALT1 (mucosa-associated lymphoid tissue lymphoma translocation gene 1). The subsequent CARD11-BCL10-MALT1 (CBM) complex can activate NF-κB, mTORC1 (mechanistic target of rapamycin complex 1), and JNK (c-Jun N-terminal kinase) and AKT pathways, thereby influencing processes such as cell survival, proliferation, and metabolism [4–7].

Distinct human pathologies result from different types of deleterious germline variants in the *CARD11* gene. The first inborn error of CARD11 function was described in 2012 when heterozygous, gain-of-function (GOF) *CARD11* variants were found to underlie a polyclonal B-cell lymphoproliferative disease termed BENTA (B-cell expansion with NF-κB and T cell Anergy) [8]. To date, approximately 30 cases of *CARD11* GOF have been reported [9]. Clinical presentation in these patients is variable, but typically includes recurrent sinopulmonary infections, susceptibility to certain opportunistic viral infections (Epstein-Barr Virus, BK virus, *Molluscum contagiosum*), poor antibody (Ab) responses to pneumococcal and polysaccharide vaccines, splenomegaly, and lymphadenopathy [8–15]. Increased incidence of malignancies and autoimmunity in some patients have also been reported [8, 13]. The first case of polyclonal B-cell lymphocytosis due to a homozygous *CARD11*^R331P^ GOF variant was recently reported [16]. Clinically, the CARD11*^R331P^* homozygous GOF patient generally resembled individuals with previously reported heterozygous *CARD11* GOF variants [16]. Both parents of the index case were heterozygous for the CARD11*^R331P^* variant, indicating this specific *CARD11* GOF variant is only pathogenic in homozygosity [16]. Importantly, somatic missense pathogenic *CARD11* variants causing GOF have been identified in several lymphoid malignancies, including diffuse large B-cell lymphoma (DLBCL), the most common form of mature B-cell lymphoma [17, 18].

By contrast, inactivating variants in *CARD11* cause other disorders. Biallelic loss-of-function (LOF) variants underlie profound combined immunodeficiency characterized by early onset, recurrent, and life-threatening infections. Since the first description in 2013, fewer than 15 cases of autosomal recessive (AR) CARD11 deficiency have been reported [19, 20], and most result in complete loss of CARD11 function. Interestingly, CARD11 deficiency does not disrupt lymphocyte development in primary lymphoid tissues, as circulating T and B cells are present in affected individuals in normal numbers. However, CARD11-deficient lymphocytes fail to progress beyond early stages of peripheral T- and B-cell maturation, evidenced by an accumulation of transitional B cells and naïve T cells, dramatic reductions in memory cells, and hypogammaglobulinemia. Consequently, AR CARD11 deficiency requires hematopoietic stem cell transplant due to compromised adaptive lymphocyte function and differentiation [21, 22]. Lastly, monoallelic *CARD11* LOF variants were first identified in 2017 in individuals with severe atopic dermatitis [6, 23]. These variants exert dominant interference on wild-type CARD11 signaling, resulting in a wider spectrum of clinical manifestations (e.g. severe atopy, recurrent respiratory/skin infections, and immune dysregulation) collectively described as CARD11-associated atopy with dominant interference of NF-κB signaling (CADINS) disease [24].

Since the initial reports of *CARD11* GOF patients, several groups have dissected the effect of these variants on various aspects of B-cell and T-cell biology using purified patient peripheral blood mononuclear cells (PBMCs), lymphocytes, cell lines, and mouse models. The poor humoral immune responses observed in many patients have been attributed to intrinsic B-cell defects such as impaired upregulation of key plasma cell-related genes (*PRDM1, CD27, CD38*) and proteins (XPB1s), and defective class switching to IgG following *in vitro* stimulation [8, 10, 12, 16]. More recently, mouse models harboring the pathogenic orthologous *Card11* C49Y CARD variant [25] and the *Card11* E134G coiled-coil domain variant (identified in three published cases [8, 10]) have enabled more comprehensive investigation of the effects of increased CARD11 activity on B-cell development and generation of T-independent immune responses [7]. The impact of *CARD11* GOF variants on signaling pathways downstream of key lymphocyte surface receptors has also been investigated in some detail [7, 8, 12, 15, 26]. However, beyond T-cell activation, the effect of these variants on the T-cell compartment remains understudied. Here, we selected a cohort of five patients from four kindreds harboring disease-causing variants in the LATCH/coiled-coil domains of CARD11 to dissect the effect on T-cell differentiation and activation and further investigate intrinsic B-cell defects. Our study confirms and substantially extends previous *in vitro* and phenotypic findings, and provides additional insight into the effects of CARD11 GOF on T-cell biology.

## Materials and methods

### Patient samples

Peripheral blood was obtained from *CARD11* GOF patients (Table 1); healthy donor buffy coats were purchased from the Australian Red Cross Blood Service (Sydney).

**Table 1:** Clinical history, genetics, and demographics of *CARD11* GOF patient cohort.

Patient	*CARD11* variant (domain)	Age (years^a^)	Sex	Infections	Organomegalies	Other manifestations	Treatment	Impaired vaccine responses	Reference
1	E134G (coiled-coil)	13	F	Recurrent sinus and middle ear infections	Splenomegaly, lymphadenopathy	—	Tonsillectomy, bilateral myringotomy tube placement	Pneumococcal, meningococcal, VZV	[9]
2	E134G (coiled-coil)	15	F	Recurrent sinus and middle ear infections	Splenomegaly	—	Tonsillectomy, adenoidectomy, bilateral myringotomy tube placement	Pneumococcal, meningococcal, VZV	[9]
3	G123S (LATCH)	13	F	Bronchitis, pneumonia, otitis media, chronic viral infections (EBV, *Molluscum contagiosum,* BK virus)	—	Auto-Abs	Bilateral myringotomy tube placement with adenoidectomy, dental extractions to remove abscesses	Pneumococcal, meningococcal, VZV	[9]
4	G123D (LATCH)	17	M	Recurrent sinusitis, middle ear infections, and bronchitis; acute EBV infection	Massive adenopathy and splenomegaly	ITP	Bilateral myringotomy tube placement, intravenous Ig, rituximab, corticosteroids, acyclovir	Tetanus, *Hemophilus influenzae*, VZV, rubeola, rubella	[11]
5	G123S (LATCH)	Mid 50s^b^	M	Suspected viral meningitis at 13 years old	Splenomegaly	Asthma	Splenectomy, chlorambucil.	ND	[27, 28]

Abbreviations: ITP, immune thrombocytopenic purpura; ND, not determined.

^a^Age given is the age of the patient at time of diagnosis or close to time of diagnosis.

^b^P5 was diagnosed with a disorder at age 2 years [26], but a genetic diagnosis of CARD11 GOF was not determined until he was in his 50s [25].

### Human antibodies

The following mAbs were used: anti-CD3 BV421, anti-CD20 BUV395, anti-CD10 APC, anti-CD4 BUV737, anti-CD4 APC-Cy7, anti-CD8 BUV395, anti-CD8 BUV496, anti-CD19 BV711, anti-CD21 BV421, anti-CD25 PECy7, anti-CD25 RY586, anti-CD27 PE, anti-CD27 PECy7, anti-CD31 BUV395, anti-CD34 FITC, anti-CD38 APC, anti-CD69 BV711, anti-CD45RA BV605, anti-CD127 BB700, anti-CCR6 PE, anti-CXCR5 A647, anti-CXCR5 BUV615-P, anti-ICOS PE-CF954, anti-PD1 BV605, anti-IgD PE, anti-IgG PE, anti-IgG BV605, anti-IgM PerCPCy5.5, Streptavidin-PerCPCy5.5, anti-IL-2 BV711, anti-IL-13 BV421, anti-IL-17F BV786, anti-IFN-γ BV605, anti-TNF-α BUV395 (all from Becton Dickinson); anti-CCR7 PECy7, anti-CD127 BV650, anti-IL-17A APCCy7, anti-CD20 Pacific Blue, anti-FoxP3 PE, anti-CXCR3 BV421 (all from BioLegend); anti-CCR7 FITC (R&D Systems); anti-IgA-biotin (SouthernBiotech); anti-IL-4 PECy7, anti-IL-21 e660, anti-IL-22 PE, anti-CD45RA PerCPCy5.5 (all from Thermo Fisher Scientific).

### Human lymphocyte phenotyping

PBMCs were labeled with mAbs against CD20, CD10, CD27, IgG, and IgA. Proportions of CD20^+^CD27^−^CD10^+^ (transitional), CD20^+^CD27^−^CD10^−^ (naïve) and CD20^+^CD27^+^CD10^−^ (memory) B cells, as well as the proportions of IgG^+^ and IgA^+^ memory B cells, were determined by flow cytometry (LSR Fortessa; Becton Dickinson) and analyzed using FlowJo software (Tree Star) as described previously [27, 28]. On transitional, naïve and memory subsets, the surface expression of CD5, CD38, IgD, and CXCR3 was determined. Analysis of T cell (CD3^+^) subpopulations determined proportions of: CD4^+^ and CD8^+^ T cells; Tfh cells; Tregs; naïve (CD45RA^+^CCR7^−^), central memory (CD45RA^−^CCR7^+^), and CD45RA^+^ or CD45RA^−^ effector memory cells within the CD4^+^ and CD8^+^ compartments as described previously [27]. CD31 expression was determined on CD4^+^ and CD8^+^ T cell subsets; CD25, CD69, and ICOS expression was determined on CD4^+^ subsets. Intracellular staining for FoxP3 was performed using the True-Nuclear Transcription Factor Buffer Set (Biolegend) according to the manufacturer's protocols. Analysis was performed on the LSRII Fortessa or FACS Symphony A5 (Becton Dickinson) and data were processed using FlowJo software v9 or v10 (TreeStar). Statistical analysis was performed using GraphPad Prism software v10.4.0.

### Isolation and functional characterization of patient B cells

PBMCs were incubated with mAbs against CD20, CD10, CD27, and IgG and washed, followed by sorting transitional (CD20^+^CD10^+^CD27^−^IgG^−^) and naïve (CD20^+^CD10^−^CD27^−^IgG^−^) B cells, using a FACSAria II or FACSAria III (Becton Dickinson), ensuring >98% purity of the recovered populations. Transitional or naïve B cells were cultured in 96-well round-bottom plates (25–30 × 10^3^ cells/well for plasmablast differentiation; 40 × 10^3^ cells/well for CFSE analysis; 5 × 10^3^ cells/well to determine Ig secretion). B cells were stimulated with 200 ng/ml CD40L cross-linked to 50 ng/ml HA Peptide mAb (R&D Systems) alone or together with different combinations of IL-4 (100 U/ml, PeproTech), IL-21 (50 ng/ml, PeproTech), the TLR agonist CpG 2006 (1μg/ml, Sigma-Aldrich), anti-IgM/G/A (2.5μg/ml, Jackson ImmunoResearch Labs), or BAFF (500 ng/ml, PeproTech). B-cell viability was determined using the Zombie Aqua Viability dye (BioLegend) and proliferation was determined by CFSE (eBioscience) as described previously [29]. Secretion of IgM, IgG, and IgA by *in vitro* cultured human transitional, naïve, and memory B cells was determined using Ig heavy-chain specific ELISAs (Southern Biotech), as described previously [29].

### Isolation and functional characterization of patient T cells

From PBMCs, naïve and memory CD4^+^ T cells were isolated by excluding Tregs (CD25^hi^CD127^lo^) and then sorting CD45RA^+^CCR7^+^ cells and CD45RA^−^CCR7^+/−^ cells, respectively. Additionally, for P5 and two healthy donors, CD4^+^ T-cell subsets were sorted according to CD45RA and CXCR5 expression. This was achieved by selecting CD3^+^CD20^−^CD4^+^ cells, excluding Tregs, then collecting the following subpopulations: naïve (CD45RA^+^CXCR5^−^CCR7^−^), memory (CD45RA^−^CXCR5^−^), Tfh-memory (CD45RA^−^CXCR5^+^), and CD45RA^+^CXCR5^+^. >98% purity of all recovered populations was confirmed. Sorted CD4^+^ T-cell subsets were cultured in 96-well round-bottom plates (30–40 × 10^3^ cells/well) with T-cell activation and expansion (TAE) beads (anti-CD2/CD3/CD28; Miltenyi Biotech) alone or together with cytokines that induce Th1 (50 ng/ml IL-12; R&D Systems) or Th17 polarization (20μg/ml IL-1β, 50 ng/ml IL-6, 200 ng/ml IL-21, 200 ng/ml IL-23, and 10 ng/ml TGF-β1; all from PeproTech) as described previously [30]. Supernatants were harvested after 5 days to measure secretion of IL-2, IL-4, IL-5, IL-13, IFN-γ, TNF-α, IL-17A, and IL-17F using cytometric bead arrays (Becton Dickinson). IL-22 secretion was measured by ELISAs (PeproTech). Additionally, on Day 5, intracellular expression of IL-2, IL-4, IL-13, IL-17A, IL-17F, IL-21, IL-22, IFN-γ, and TNF-α was analyzed by flow cytometry following 6 hours of re-stimulation of cells with PMA and ionomycin, including addition of Brefeldin A (10μg/ml) after 2 hours [30].

### Assessment of phosphorylation of S6 kinase

PBMCs from healthy donors and CARD11 GOF patients were thawed, rested overnight, and T cells were then activated with 1 mg/ml soluble anti-CD3/CD28 mAb for 3 days (clones: HIT3a/CD28.2, BD Biosciences). After this time, T cells were placed in complete RPMI1640 media (RPMI1640/10% fetal bovine serum/1% penicillin/streptomycin; Millipore Sigma) with 100 U/ml rIL-2 (Thermo Scientific) and expanded for ∼10 days before cryopreservation. Effector T cells were thawed, rested overnight, then plated at 5 × 10^5^ cells/ml in 1 ml of complete RPMI in a 24-well plate and then cultured either in media alone (unstimulated) or in the presence of 1 μg/ml soluble anti-CD3/CD28 mAbs. After 24 hours, cells were collected for flow cytometric staining and analysis. Cells were live/dead stained with Zombie NIR (Biolegend: 423105). The surface staining panel contained anti-CD4 (BD Biosciences; fluorophore: BUV496; clone: SK3), anti-CD8 (BD Biosciences; fluorophore: BUV805; clone: SK1), and anti-CD69 (Biolegend; fluorophore: BV750; clone: FN50) mAbs. Cells were fixed/permed with the CytoFast Fix/Perm Buffer Kit (Biolegend: 426803) and stained intracellularly with anti-pS6 mAb (clone: D57.2.2E). Flow cytometry data were collected on a 5-laser Cytek Aurora. Flow cytometry data were analyzed using FlowJo. Gating strategy is as follows: lymphocytes → singlets → live → CD4/CD8 → CD69+ → pS6^+^. MFI was calculated using the positive pS6 gate.

## Results

### CARD11 GOF patients

To date, >30 *CARD11* GOF patients have been identified globally, with most of the pathogenic variants clustering in the coiled coil and LATCH domains of the CARD11 protein [9]. Within our cohort of five patients, three different germline *CARD11* variants are represented, all localizing to these common domains (Table 1). P1–P4 were classified as pediatric at the time of sample collection; all patients have been published previously [8, 10, 31, 32]. Four of the five individuals had a history of recurrent respiratory and/or middle ear infections, and poor Ab responses to pneumococcal, meningococcal, or other vaccines. Severe viral infections were noted in at least two patients, with EBV being commonly implicated (Table 1).

### Phenotypic characterization of *CARD11* GOF B cells

**Figure 1:**
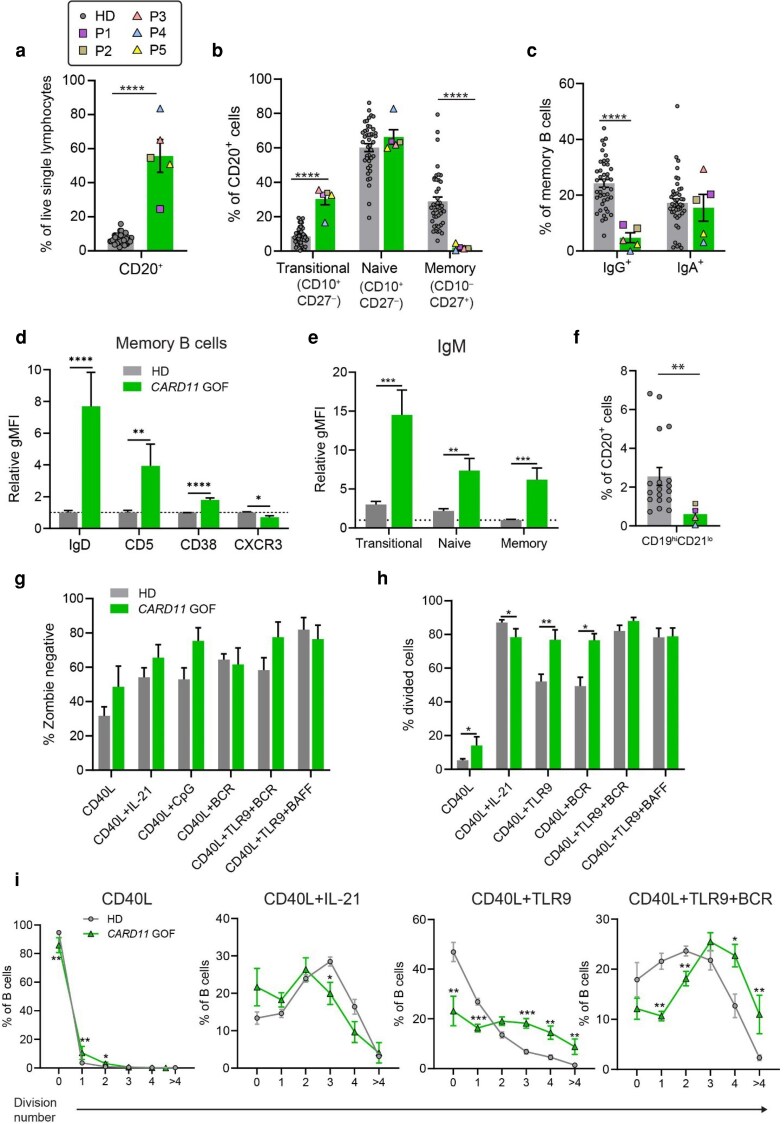
*CARD11* GOF mutations impair B-cell development but enhance B-cell proliferation. Peripheral blood mononuclear cells (PBMC) from HDs (n = 18–43) and *CARD11* GOF patients (n = 4–5) were analyzed by flow cytometry to determine percentages of (a) total B cells (CD20^+^); (b) transitional (CD10^+^CD27^−^), naïve (CD10^−^CD27^−^) and memory (CD10^−^CD27^+^) B cells; (c) IgG^+^ or IgA^+^ switched memory B cells. (d and e) Expression of (d) IgD, CD5, CD38, and CXCR3 on memory B cells; and (e) IgM on transitional, naïve, and memory B cells was also determined. Graphs show relative geometric mean fluorescence intensity (gMFI) normalized to the average of HD memory B cells within each independent experiment. HD datapoints include age-matched individuals: 16–22 years [27]. (f) Percentages of CD19^hi^CD21^lo^ B cells were also determined. Each symbol in the graphs presented in panels (a–c) and (f) represents data from an individual HD or a CARD11 GOF patient, denoted by the indicated symbols. (g–i) Naïve B cells from HDs (n = 17–18) and *CARD11* GOF patients (n = 5) were CFSE-labeled and cultured with CD40L alone or together with BCR (anti-Ig) or TLR9 (CpG) agonists or IL-21. Cells were harvested after ∼4 days. (g) B-cell viability determined by Zombie exclusion. (h) % total divided cells under each culture condition were determined by CFSE dilution. (i) % cells in each division interval. Data compiled from five independent experiments. Bars represent SEM. Statistical significance was determined by Mann–Whitney tests, *P < 0.05, **P < 0.01, ***P < 0.001, ****P < 0.0001.

Since the initial description of human germline *CARD11* GOF variants, several studies have focused on elucidating B-cell defects that may underlie poor vaccine responses and frequent life-threatening susceptibility to respiratory infections in these patients [8, 10, 12]. Flow cytometric analysis of five *CARD11* GOF individuals confirmed substantially increased percentages of circulating peripheral B cells (CD20^+^, Fig. 1a). This was not age-related, as even the *CARD11* GOF adult patient had a significantly higher frequency of peripheral B cells compared to healthy donors (HD; Fig. 1a). Despite this increase, peripheral B-cell maturation was severely compromised, evidenced by a significant increase in percentages of transitional (CD10^+^CD27^−^) B cells and a concurrent reduction in memory B cells (Fig. 1b). Assessment of Ig class switching *ex vivo* by *CARD11* GOF memory B cells also found significant reductions in percentages of IgG^+^ cells compared to memory B cells present in HD (Fig. 1c). Interestingly, and in contrast to class switching to IgG, percentages of IgA^+^ memory B cells were not affected by *CARD11* GOF variants (Fig. 1c).

To explore this maturation block in more detail, we assessed expression of surface receptors that are either typically downregulated (IgM, IgD, CD5, CD38) or upregulated (CXCR3) as transitional B cells mature into naïve and then memory B cells [33–35]. *CARD11* GOF patient memory B cells expressed much higher levels of IgD (8-fold), IgM (4 to 6-fold), CD5 (4-fold) and CD38 (2-fold), and low levels of the chemokine receptor CXCR3 than observed on corresponding memory B cells from HD (Fig. 1d and e). Thus, despite acquiring expression of CD27, the few memory B cells detected in *CARD11* GOF patients exhibited a surface phenotype more similar to transitional or naive B cells rather than mature memory B cells.

Lastly, we quantified ‘atypical’ CD21^lo^CD19^hi^ B cells, a subset that is expanded in several inborn errors of immunity—such as STAT3 GOF, CTLA4 haploinsufficiency, and autosomal recessive LRBA deficiency [36]—and implicated in immune dysregulatory states including chronic infection, autoimmunity, and lymphadenopathy [37, 38]. CD21^lo^CD19^hi^ B cells represent ∼2.5% (range: 0.5–7%) of B cells in the peripheral blood of HD (Fig. 1f). Percentages of these cells were significantly reduced in *CARD11* GOF patients, comprising <1% of circulating B cells (Fig. 1f).

### *CARD11* GOF variants have opposing effects on B-cell proliferation and differentiation

Signaling through the mTOR and NF-κB pathways has been well established to regulate cell survival, proliferation, and differentiation [39–41], thus implicating CARD11 in these processes, as it is known to regulate these signaling axes. Previous studies showed that B cells from some *CARD11* GOF patients undergo normal or enhanced proliferation *in vitro* following stimulation through the BCR, CD40, and/or BAFF-R in combination with cytokines such as IL-21, IL-4, or IL-10 [8, 12]. When we performed similar experiments, we found that *CARD11* GOF B cells exhibited similar survival rates *in vitro* as B cells from HDs, indicated by comparable levels of zombie viability dye exclusion (Fig. 1g). However, and consistent with previous data [8, 12], we found that purified naïve *CARD11* GOF B cells stimulated with CD40L alone or in combination with Toll-like receptor (TLR)9 or BCR agonists for 4 days underwent more proliferation than naïve B cells from HD (Fig. 1h and i). When stimulated with CD40L for 4 d, approximately 15% of patient B cells had divided at least once compared with only ∼5% of HD B cells (Fig. 1h and i). This proliferative advantage became more pronounced when B cells were additionally stimulated through TLR9 (using CpG) or the BCR (using anti-IgM/IgG/IgA). Under both conditions, nearly 80% of *CARD11* GOF B cells underwent at least one division compared to only ∼50% of HD naïve B cells (Fig. 1h). In the CD40L + TLR9 culture condition, analysis of individual divisions revealed that significantly more naïve *CARD11* GOF B cells that had undergone proliferation were in the 3rd or 4th division interval compared to HD, where most divided B cells were in the 1st or 2nd interval. Contrasting this, proliferation induced by CD40L and IL-21 was modestly, but significantly, reduced for *CARD11* GOF patient B cells compared to HD naïve B cells (Fig. 1h and i).

**Figure 2:**
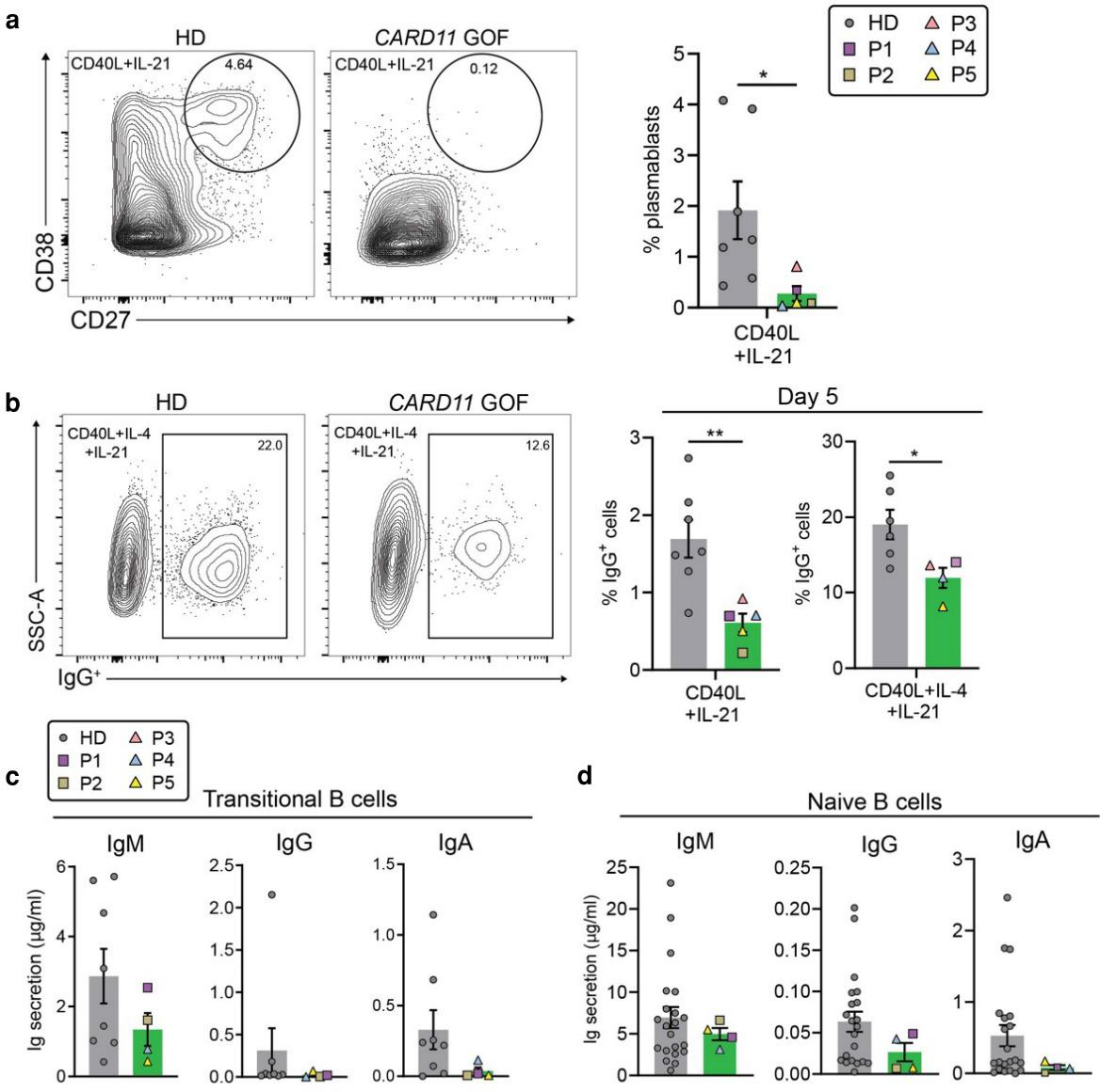
*CARD11* GOF B cells exhibit defective differentiation, Ig class-switching, and Ig secretion *in vitro*. (a and b) Naïve B cells isolated from HDs (*n* = 7) and *CARD11* GOF patients (*n* = 5) were cultured for 5 days with CD40L/IL-21 without or with IL-4. Cells were harvested and percentages of (a) plasmablasts (CD27^hi^CD38^hi^) and (b) IgG^+^ switched cells were then determined by flow cytometry. FACS plots show gating on a representative HD and *CARD11* GOF patient; summary data are from 4 to 5 independent experiments. (c) Transitional and (d) naïve B cells isolated from HDs (*n* = 8–21) and *CARD11* GOF patients (*n* = 4) were cultured for 7 days with CD40L and IL-21. ELISAs were performed on culture supernatants to quantify IgM, IgG, and IgA secretion. Each symbol in the graphs represents data from an individual HD or a CARD11 GOF patient, denoted by the indicated symbols. Bars represent SEM. Statistical significance was determined by Mann–Whitney tests, *P < 0.05, **P < 0.01.

Next, B-cell differentiation was investigated by assessing the generation of Ab-secreting cells and induction of Ig class switching *in vitro*. Following culture with CD40L and IL-21 for 5 d, naïve B cells from HD give rise to a small but detectable population of both CD38^hi^CD27^hi^ plasmablasts (Fig. 2a) and IgG^+^ class-switched B cells (Fig. 2b), the latter of which could be significantly increased (∼10-fold) by the addition of IL-4 (Fig. 2b) [42, 43]. Strikingly, differentiation of *CARD11* GOF naïve B cells into plasmablasts or IgG-switched cells was significantly decreased compared with naïve B cells from HD (Fig. 2a and b). While CD40L/IL-21-stimulated *CARD11* GOF B cells were responsive to IL-4, the percentage of IgG^+^ cells formed in response to this condition (i.e. CD40L/IL-21/IL-4) was still significantly reduced compared to HD naïve B cells (Fig. 2b). These results are consistent with previous studies, which used a combination of anti-CD40, IL-21, anti-IgM and IL-2 to examine plasma cell differentiation and class-switching in B cells from *CARD11* GOF patients [12].

To complement the above findings, we examined Ig secretion by sort-purified transitional and naïve B cells after 7 days of stimulation with CD40L and IL-21. *CARD11* GOF transitional B cells showed a 2-fold reduction in IgM secretion and greatly impaired production of IgG and IgA compared to HD transitional B cells (Fig. 2c). Interestingly, *CARD11* GOF naïve B cells showed intact IgM secretion. However, levels of IgG and IgA were decreased 2-fold and 4-fold, respectively (Fig. 2d), consistent with previous findings [10, 12]. Combined, our data confirm at the cellular, phenotypic, and functional levels that *CARD11* GOF B cells exhibit a significantly impaired ability to undergo plasmablast differentiation and Ig class switching *in vitro*. Importantly, this was observed for *CARD11* GOF B cells at both the transitional and naïve stages of maturation.

### *CARD11* GOF alters phenotype and function of CD4^+^ T cells

**Figure 3:**
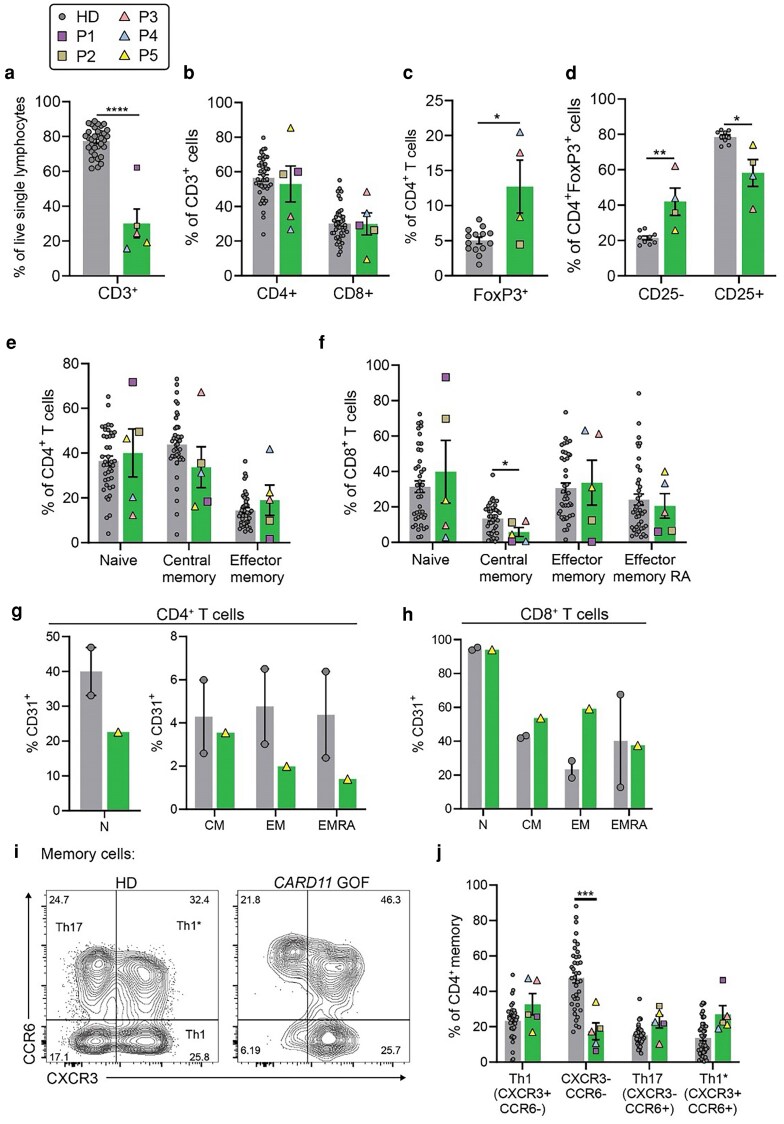
Phenotypic characterization of CD4^+^ and CD8^+^ T-cell compartments in *CARD11* GOF. (a–d) PBMCs from HDs (*n* = 9–41) and *CARD11* GOF (*n* = 5) patients were analyzed by flow cytometry to determine % of (a) total T cells (CD3^+^); (b) CD4^+^ and CD8^+^ T cells; (c) regulatory T cells (FoxP3^+^); and (d) CD25^−^ and CD25^+^ cells within the Treg population. (e–h) Within (e and g) CD4^+^ and (f and h) CD8^+^ T-cell populations, % of (e and f) naïve (CD45RA^+^CCR7^+^), T_CM_ (CD45RA^−^CCR7^+^), T_EM_ (CD45RA^−^CCR7^−^), and T_EMRA_ (CD45RA^+^CCR7^−^) cells, as well as (g and h) recent thymic emigrants (CD31^+^) in CD4^+^ and CD8^+^ T-cell subpopulation were determined for HDs and *CARD11* GOF P5. (e and f): HD (*n* = 41), *CARD11* GOF (*n* = 5). (g and h): HD (*n* = 2), *CARD11* GOF (n = 1) (P5). HD datapoints include age-matched individuals: 16–22 years [27]. (i and j) Total memory CD4^+^ T cells (CD45RA^−^CXCR5^−^) present in peripheral blood of HDs (*n* = 38) and *CARD11* GOF (*n* = 5) were analyzed based on differential expression of CXCR3 and CCR6 to determine percentages of T helper (Th)1-like (CXCR3^+^CCR6^−^), CXCR3^−^CCR6^−^, Th17-like (CXCR3^−^CCR6^+^), and Th1* (CXCR3^+^CCR6^+^) cells. Bars represent SEM. Statistical significance was determined by Mann–Whitney tests, *P < 0.05, ***P < 0.001, ****P < 0.0001. Each symbol in the graphs presented in panels (a–f) and (j) represents data from an individual HD or a CARD11 GOF patient, denoted by the indicated symbols.

Patients harboring *CARD11* GOF mutations have reduced percentages but normal numbers of peripheral blood T cells [8, 10, 11]. We verified this for the patient cohort in the present study, establishing that percentages of total CD3^+^ T cells were significantly reduced but those of CD3^+^CD4^+^ and CD3^+^CD8^+^ T cells were within the range of HD (Fig. 3a and b). Interestingly, within our cohort of *CARD11* GOF patients, several had increased percentages of T regulatory cells (Tregs), defined as CD4^+^FoxP3^+^ cells (Fig. 3c). Furthermore, fewer *CARD11* GOF CD4^+^FoxP3^+^ cells were CD25^+^ compared to Tregs in HD (Fig. 3d). Further studies are required to assess the functionality of these distinct subsets of Tregs both in HD and CARD11 GOF patients.

Delineating subsets of CD4^+^ and CD8^+^ T cells according to differential expression of CD45RA and CCR7 [44] revealed comparable percentages of naïve (CD45RA^+^CCR7^+^) and effector memory (T_EM_, CD45RA^−^CCR7^−^) CD4^+^ and CD8^+^ T cells, as well as of CD4^+^ central memory (T_CM_, CD45RA^−^CCR7^+^) and CD8^+^ T_EMRA_ (CD45RA^+^CCR7^−^) in HD and *CARD11* GOF patients (Fig. 3e and f). The only T-cell subset affected was CD8^+^ T_CM_ cells, which were significantly reduced in *CARD11* GOF patients (Fig. 3f). However, there was a wide range in naïve and T_EM_ percentages when comparing individual patients. While two out of five patients had a history of EBV infection (Table 1), increased frequencies of CD8^+^ T_EMRA_ within our cohort did not correlate with patients’ EBV infection status as might be expected [45, 46].

To assess T-cell development and differentiation further, we examined surface expression of CD31, which defines recent thymic T-cell emigrants [47]. Patients’ naïve, T_CM_, T_EM_, and T_EMRA_ subsets within the CD4^+^ and CD8^+^ T-cell populations contained comparable percentages of CD31^+^ cells to HD, suggesting *CARD11* GOF variants do not alter thymic egress (Fig. 3g and h).

Differential expression of CXCR3 and CCR6 delineates CXCR3^+^CCR6^−^ T helper (Th)1, CXCR3^−^CCR6^+^ Th17, and CXCR3^+^CCR6^+^ Th1* cells within the memory compartment (CD45RA^−^CXCR5^−^) of CD4^+^ T cells [48, 49]. Applying this analysis showed a reduction in percentages of CXCR3^−^CCR6^−^ memory CD4^+^ T cells—which are enriched for Th2 and Th9 cells [30, 48, 50]—and modest but not significant increases in percentages of Th1, Th17, or Th1* memory T cells in *CARD11* GOF patients compared to HD (Fig. 3i and j). Circulating Tfh-type cells (cTfh; CD45RA^−^CXCR5^+^) [30, 48] similarly showed a 2-fold decrease in CXCR3^−^CCR6^−^ cells, and a modest increase in CXCR3^+^CCR6^+^ cells (data not shown).

### *CARD11* GOF variants enhance proliferation but impair production of Th2 cytokines by CD4^+^ T cells

**Figure 4:**
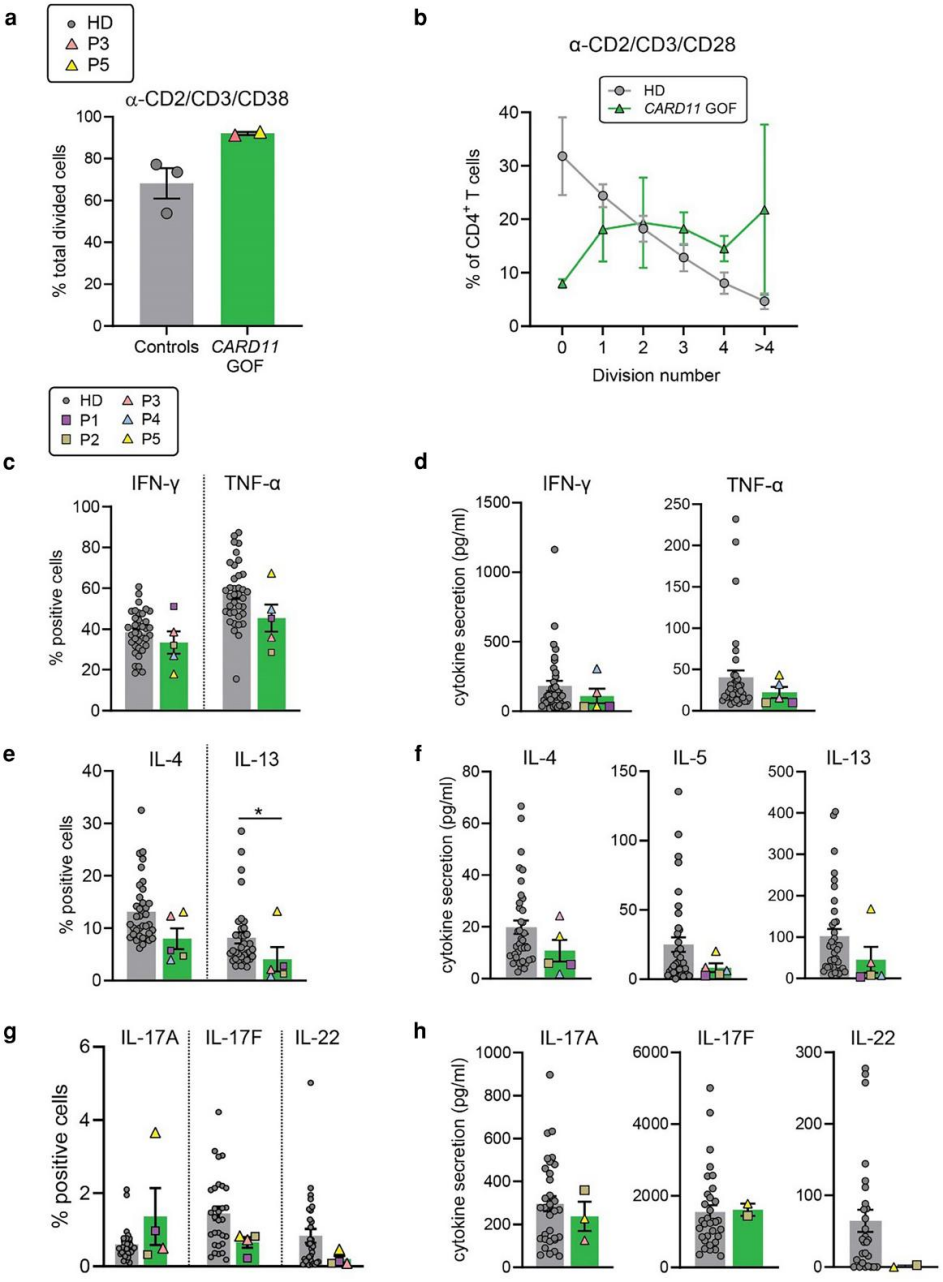
Effect of *CARD11* GOF variants on CD4^+^ T-cell function. (a and b) Naïve CD4^+^ T cells from HDs (*n* = 3) and *CARD11* GOF patients (*n* = 2) were CFSE-labeled and cultured for 5 days with TAE beads to determine proliferation. Percentage of (a) total divided cells and (b) cells in each division interval. Not statistically significant by Mann–Whitney tests. (c–h) Memory CD4^+^ T cells from HDs (*n* = 28–37) and *CARD11* GOF patients (*n* = 3–5) were cultured for 5 days with TAE beads alone (Th0 conditions) to induce IFN-γ, TNF-α, IL4, IL-5, and IL-13 production (d–f), or under Th17-polarizing conditions to amplify IL-17A, IL-17F, and IL-22 production (g and h). Intracellular expression and secretion of cytokines associated with (c and d) Th1, (e and f) Th2, and (g and h) Th17 subsets were determined by intracellular staining (c, e and g) and cytometric bead array (d, f and h), respectively. Each symbol in the graphs presented in panels (a–f) and (j) represents data from an individual HD or a CARD11 GOF patient, denoted by the indicated symbols. Bars represent SEM. Statistical significance was determined by Mann–Whitney tests, *P < 0.05.

Functional studies of murine and human *CARD11* GOF T cells have found impaired activation and proliferation of total T cells cultured with anti-CD3/CD28 mAbs; however, this could be rescued by supplementing the cultures with anti-CD2 mAb or exogenous IL-2 [8, 51]. To investigate proliferation within our *in vitro* culture system, naïve CD4^+^ T cells were sort-purified from *CARD11* GOF patients and HD, labeled with CFSE and cultured for 4 d with anti-CD2/CD3/CD28 mAb T cell activation and expansion (TAE) beads. Under the experimental conditions used in our study, naive CD4^+^ T cells from *CARD11* GOF patients underwent greater proliferation than naive CD4^+^ T cells from HD (Fig. 4a and b).

Cytokine production by memory CD4^+^ T cells was then assessed after 5 days of stimulation with TAE beads (Th0), followed by restimulation with phorbol myristate acetate (PMA) and ionomycin. Intracellular expression and secretion of the T helper (Th)1-associated cytokines IFN-γ and TNF-α by *CARD11* GOF memory CD4^+^ T cells did not significantly differ from that of HD memory CD4^+^ T cells (Fig. 4c and d). In contrast, there was a ∼2-fold reduction in expression of Th2 cytokines (IL-4, IL-13 [*P* < 0.05]) by *CARD11* GOF memory CD4^+^ T cells compared to HDs (Fig. 4e). Consistent with this, secretion of IL-4, IL-5, and IL-13 by memory *CARD11* GOF CD4^+^ T cells was also 2-fold less than HD memory CD4^+^ T cells (Fig. 4f), however IL-9 production was intact (not shown) providing additional evidence that Th2 and Th9 cells are distinct and have unique requirements for their generation and function [50]. This establishes a Th2 defect and is consistent with the paucity of CD4^+^CD45RA^−^CXCR3^−^CCR6^−^ Th2-phenotype cells in peripheral blood of *CARD11* GOF patients (Fig. 3j). Interestingly, following stimulation under Th17-polarizing conditions, production of IL-17A and IL-17F by *CARD11* GOF memory CD4^+^ T cells was generally comparable to HD (Fig. 4g and h). However, there was a trend towards reduced IL-22 production, especially when secretion was assessed (Fig. 4g and h). Thus, *CARD11* GOF mutations appear to selectively impair cytokine production, especially Th2-type cytokines.

### *CARD11* GOF patients have an expanded population of CD4^+^CD45RA^+^CXCR5^+^ T cells

**Figure 5:**
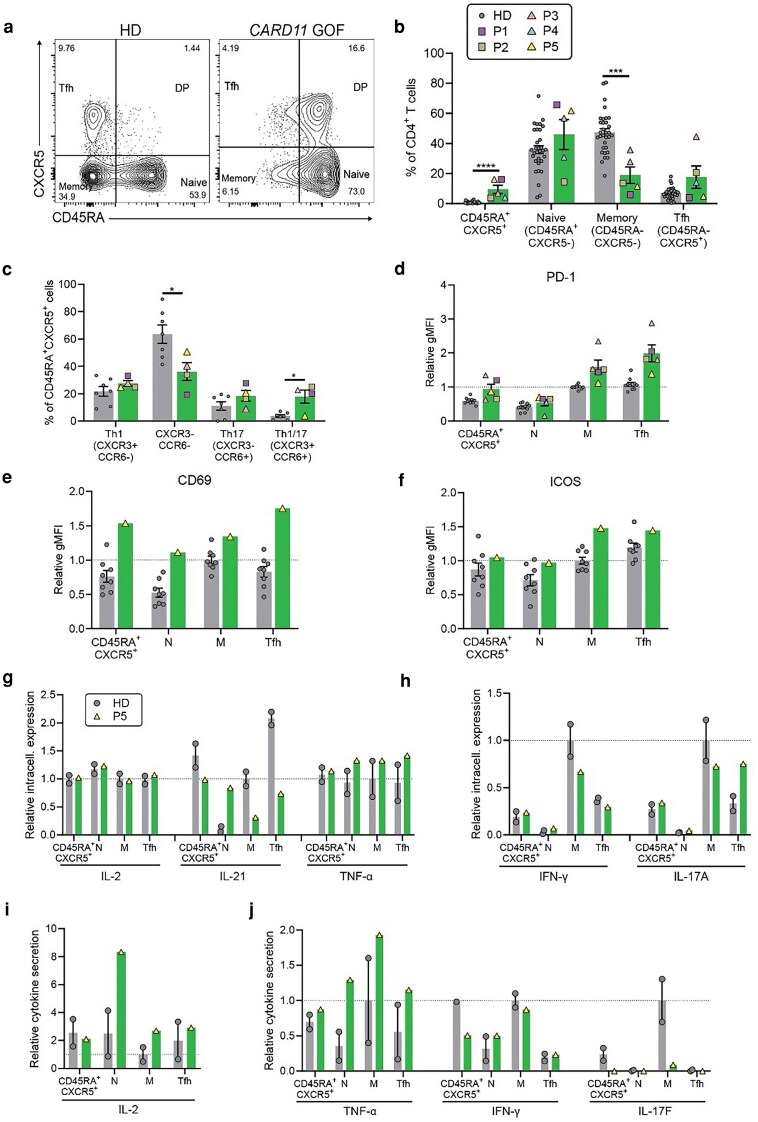
CD4^+^ T cells from *CARD11* GOF patients contain a distinct subset of CD45RA^+^CXCR5^+^ cells. (a–c) PBMCs from HDs (*n* = 7–32) and *CARD11* GOF patients (*n* = 4–5) were analyzed to determine percentages of naïve (N; CD45RA^+^CXCR5^−^CCR7^+^), memory (M; CD45RA^−^CXCR5^−^), circulating T follicular helper (cTfh; CD45RA^−^CXCR5^+^), or CD45RA^+^CXCR5^+^ CD4^+^ T cells. (a) Gating on CD4^+^ T cells from a representative HD and *CARD11* GOF patient. (c) % Th1-like (CXCR3^+^CCR6^−^), CXCR3^−^ CCR6^−^, Th17-like (CXCR3^−^CCR6^+^), and Th1* (CXCR3^+^CCR6^+^) cells in CD45RA^+^CXCR5^+^ T-cell subset. For graphs depicted in panels (b) and (c), HD datapoints include age-matched individuals: 16–22 years [27]. (d–f) Expression of (d) PD-1, (e) CD69, and (f) ICOS on naïve, memory, cTfh, and CD45RA^+^CXCR5^+^ CD4^+^ T-cell subsets. The graph shows relative gMFI normalized to the average of HD memory CD4^+^ T cells within each independent experiment. Data were combined from three independent experiments. (g–j) Sorted naïve, memory, Tfh, and CD45RA^+^CXCR5^+^ cells from HDs (*n* = 2) and *CARD11* GOF P5 (*n* = 1) were cultured for 5 days with TAE beads alone to induce IL-2, IL-21, TNF-α, IFN-γ production, IL-17A, and IL-17F production. (g and h) Intracellular cytokine expression was determined by flow cytometry. (i and j) Cytokine secretion was determined cytometric bead arrays. Graphs show the relative frequency of cytokine-positive cells or amount of cytokine secreted, normalized to the average of memory cells from the HDs within each independent experiment. Each symbol in the graphs represents data from an individual HD or a CARD11 GOF patient, denoted by the indicated symbols. Bars represent SEM. Statistical significance was determined by Mann–Whitney tests, *P < 0.05, ***P < 0.001, ****P < 0.0001.

While comparable frequencies of CD4^+^ T-cell subsets were observed in *CARD11* GOF patients and HD when defined by differential expression of CD45RA and CCR7 [44], defining CD4^+^ T-cell subsets based on CD45RA and CXCR5 expression revealed marked differences. *CARD11* GOF individuals had greatly increased percentages of cells co-expressing CD45RA and CXCR5. Such cells are rare in HD but represented a distinct population within the total CD4^+^ T-cell pool of *CARD11* GOF patients (Fig. 5a and b; HD: 0.84 ± 0.11% of CD4^+^ T cells, *n* = 32; patients: 9.4 ± 2.8%, *n* = 5; mean ± SEM). As B cells also express CXCR5, we confirmed that this population was not derived from abundant B cells within the stained patient PBMC sample, by simultaneously staining with the pan-B cell marker CD20. This confirmed that the CD4^+^CD45RA^+^CXCR5^+^ cells were not doublets of B cells and CD4^+^ T cells (data not shown). Non-Tfh memory CD4^+^ T cells (CD45RA^−^CXCR5^−^) were reduced >2-fold in *CARD11* GOF individuals (HD: 47.4 ± 2.5%, *n* = 32; patients: 19.0 ± 5.5%, *n* = 5; mean ± SEM) (Fig. 5b), while percentages of naïve (CD45RA^+^CXCR5^−^CCR7^+^) and cTfh (CD45RA^−^CXCR5^+^) cells were unchanged between *CARD11* GOF patients and HD (Fig. 5b). Further analyses revealed that the CD45RA^+^CXCR5^+^ CD4^+^ T-cell population in *CARD11* GOF patients contained a similar distribution of Th1 and Th17 cells as memory (CD45RA^−^CXCR5^−^) and CXCR5^+^ cTfh subsets in the patients (compare Fig. 5c and Fig. 3j; data not shown). In both subsets, percentages of CXCR3^−^CCR6^−^ cells were reduced and CXCR3^+^CCR6^+^ cells generally increased compared to HD (Fig. 5c).

CXCR5 is induced on CD4^+^ T cells following engagement of the T-cell receptor [52, 53]. Given the higher percentage of CD45RA^+^CXCR5^+^ cells observed in the CD4^+^ T-cell compartment of multiple *CARD11* GOF patients (Fig. 5b), we hypothesized that *CARD11* GOF CD4^+^ T cells may exhibit an increased state of basal activation. To assess this, we determined expression of other activation markers on CD4^+^ T cells *ex vivo*, including the early activation marker CD69, and PD-1 and ICOS, which are highly expressed on activated T cells—particularly Tfh cells—in human lymphoid tissues [54, 55]. With respect to peripheral blood CD4^+^ T-cell subsets in HD, levels of PD-1 and ICOS are markedly increased on memory and cTfh cells compared to naïve and CD45RA^+^CXCR5^+^ T cells, while expression of CD69 is low on all HD CD4^+^ T-cell subsets (Fig. 5d–f). Expression of PD-1 on *CARD11* GOF CD4^+^ T-cell subsets from all patients, as well as ICOS on P5's CD4^+^ T cells, followed the same pattern as CD4^+^ T cells from HD; however, the absolute levels detected on *CARD11* GOF CD45RA^+^CXCR5^+^ and cTfh cell subsets were 1.5–2.5-fold higher than corresponding subsets from HD (Fig. 5d and f). Analysis of P5 also revealed CD69 expression on all CD4^+^ T-cell subsets, including naïve cells, which was up to 2-fold higher than on corresponding CD4^+^ T-cell subsets in HD (Fig. 5e). Taken together, these data suggest that CD45RA^+^CXCR5^+^ CD4^+^ T cells present in HD and *CARD11* GOF patients exhibited a phenotype intermediate to that of naïve and memory/cTfh cells. Thus, *CARD11* GOF may result in increased basal T-cell activation, indicated by acquisition of a surface phenotype consistent with constitutive basal TCR signaling. Similarly, mice harboring a *CARD11* GOF mutation that is recurrently found in DLBCL have increased percentages of activated T cells, Tfh cells, and Tregs, each expressing higher levels of ICOS and PD-1 as well as other activation markers [51].

To assess the functional capacity of the CD4^+^CD45RA^+^CXCR5^+^ subset, naïve (CD45RA^+^CXCR5^−^ CCR7^+^), memory (CD45RA^−^CXCR5^−^), cTfh (CD45RA^−^CXCR5^+^), and CD45RA^+^CXCR5^+^ CD4^+^ T cells were sort-purified from HDs and patient 5 (P5), then cultured with TAE beads for 5 days to examine cytokine production. Overall, intracellular expression of IL-2, Th1 cytokines (TNF-α, IFN-γ), and IL-17A was comparable between P5 and HD, while IL-21 was lower in *CARD11* GOF memory and cTfh cells (Fig. 5g and h). Similar to the trend in expression of activation markers (Fig. 5d–f), cytokine production by CD4^+^CD45RA^+^CXCR5^+^ T cells from the *CARD11* GOF patient and HD was generally intermediate to naïve CD4^+^ T and memory/cTfh cells (Fig. 5g and h). Consistent with this, CD4^+^CD45RA^+^CXCR5^+^ T cells from HD secreted higher levels of TNF-α, IFN-γ, and IL-17F than naïve cells (Fig. 5i and j). However, there were no discernible differences in cytokine secretion between this CD4^+^ T-cell subset in a *CARD11* GOF patient compared to HDs (Fig. 5i and j).

### CARD11 GOF T cells exhibit increased mTOR/PI3 kinase signaling

**Figure 6:**
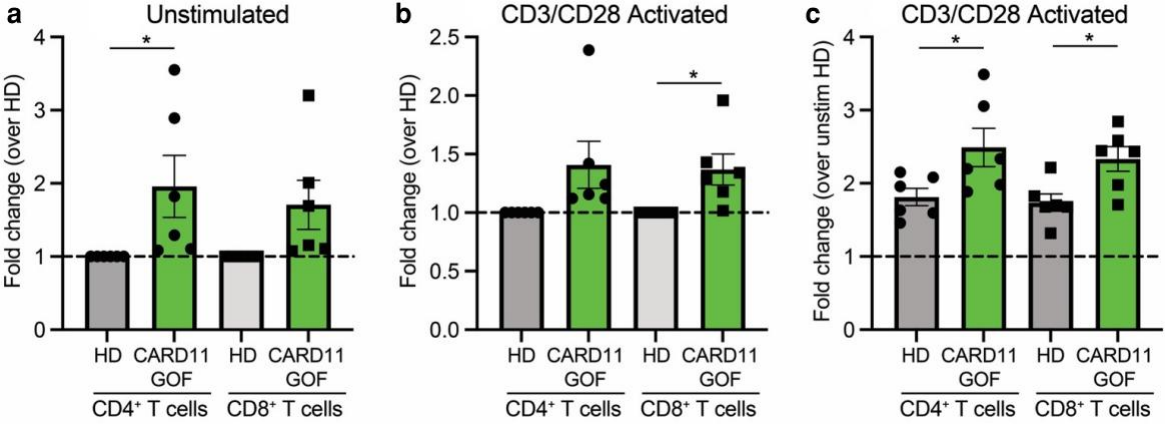
Increased PI3K/mTOR signaling in *CARD11* GOF T cells. T-cell lines established from healthy donors (HD) or CARD11 GOF patients were either unstimulated or activated with anti-CD3/CD28 mAbs for 24 hours. After this time, cells were harvested and levels of phospho-S6 in CD4^+^ and CD8^+^ T cells were quantified by surface and intracellular flow cytometric staining using mAbs specific for CD4, CD8, CD69, and pS6. The MFI of pS6 was determined for CD4^+^CD69^+^ or CD8^+^CD69^+^ T cells. Data are combined from two independent experiments that used T cells from five different HD or 3 CARD11 GOF. Data depict mean ± SEM fold change of pS6 MFI in (a) unstimulated CARD11 GOF CD4^+^ or CD8^+^ T cells relative to unstimulated HD CD4^+^ and CD8^+^ T cells; (b) stimulated CARD11 GOF CD4^+^ or CD8^+^ T cells relative to stimulated HD CD4^+^ and CD8^+^ T cells; or (c) stimulated CARD11 GOF CD4^+^ or CD8^+^ T cells relative to unstimulated HD CD4^+^ and CD8^+^ T cells. Statistical significance was determined by unpaired *t* tests. *P < 0.05, ***P < 0.001, ****P < 0.0001.

CARD11 can regulate activation of the NF-κB, mTORC1, JNK, and AKT pathways [4–7]. To investigate this further, we assessed phosphorylation of the ribosomal S6 protein (pS6), which is downstream of PI3K/mTOR/AKT signaling pathways, in T cell lines established from HDs and CARD11 GOF patients. Basal levels of pS6 were 1.7–2-fold increase in CARD11 GOF CD4^+^ and CD8^+^ T cells compared to corresponding T cell lines from HDs (Fig. 6a). Heightened levels of pS6 continued to be observed in CARD11 GOF T cells following *in vitro* stimulation in response to anti-CD3/CD28 mAbs (∼1.4-fold, Fig. 6b). The augmented levels of pS6 in CARD11 GOF CD4^+^ and CD8^+^ T cells were also evident when the data for stimulated T cells are expressed as fold change compared to unstimulated HD T cells (Fig. 6c). These data clearly establish increased mTOR/PI3K/Akt activation in CARD11 GOF T lymphocytes, consistent with heightened basal T-cell activation.

## Discussion

Studies of patients with pathogenic *CARD11* GOF variants have predominantly focused on understanding effects on B-cell biology. Here, we have confirmed profound defects in human B-cell development and function due to CARD11 GOF. These include an impressive accumulation of transitional B cells and a concomitant reduction of total memory and IgG^+^ memory B cells. Furthermore, the phenotype of transitional, naïve, and memory B-cell subsets in *CARD11* GOF patients indicated they were less mature than corresponding B-cell subsets in HDs. This was further evidenced at a functional level, with rates of plasmablast differentiation, Ig class switching, and Ab secretion *in vitro* by transitional and naïve *CARD11* GOF B cells being greatly reduced compared to HD B cells, consistent with B-cell developmental and functional arrest. Curiously, *CARD11* GOF naïve B cells exhibited enhanced proliferation *in vitro* in response to various signaling inputs. An extension of this observation is that—despite enhanced cell division potentially enabling B cells to undergo greater levels of class switching and differentiation into Ab-secreting cells [42, 56]—greater proliferation of *CARD11* GOF B cells was not beneficial for B-cell effector function. These observations likely contribute to both the polyclonal B-cell lymphocytosis and impaired humoral immunity that are characteristic of these patients.

The limited investigations of *CARD11* GOF patients’ T cells to date identified anergic T-cell responses *in vitro,* including diminished Ca^2+^ flux, activation, and proliferation in response to TCR co-stimulation, which could be corrected by supplementing with anti-CD2 mAb [8]. Our *in vitro* analyses of *CARD11* GOF CD4^+^ T cells used anti-CD2/CD3/CD28 beads alone or together with T helper-polarizing cytokines. This approach demonstrated that proliferation, as well as IL-2 secretion and expression, were intact under these conditions, with a trend of enhanced T cell expansion. Interestingly, *ex vivo* phenotyping of *CARD11* GOF patients’ CD4^+^ T cells revealed aberrant distribution of T-helper subsets and production of associated cytokines. Reductions in CXCR3^−^CCR6^−^ Th2-phenotype cells were observed within the memory (CD45RA^−^CXCR5^−^) and cTfh (CD45RA^−^CXCR5^+^) subsets, while the percentage of CXCR3^+^CCR6^+^ cells was increased. The CXCR3^−^CCR6^−^ population contains Th2 and Th9 cell subsets of CD4^+^ T cells [30, 57], while CXCR3^+^CCR6^+^ cells are thought to represent a population with features of both Th1 and Th17 cells [58]. Patients’ memory CD4^+^ T cells expressed and secreted equivalent levels of Th1 (IFNγ, TNF), Th9 (IL-9), and Th17 (IL-17A, IL-17F) cytokines compared to HD cells. However, intracellular expression and secretion of Th2-associated cytokines by *CARD11* GOF memory CD4^+^ T cells were significantly reduced. This latter finding complements previous observations of enhanced Th2 responses in mice and humans with pathogenic hypomorphic, dominant negative variants that reduce CARD11 activity [6, 23, 59, 60]. For instance, memory CD4^+^ T cells from a heterozygous *CARD11* LOF patient showed elevated IL-4 and IL-13 production following *in vitro* stimulation [23]. Together, this provides further evidence that CARD11 is a key regulator of Th2 responses. Indeed, it was recently shown that initial skewing toward a Th2 fate in heterozygous *CARD11* LOF patients is likely caused by impaired JNK signaling downstream of CARD11, leading to accumulation of GATA3, the master transcription factor governing Th2 cell differentiation [6]. Th2 cytokine deficiency could also be contributing to the paucity of IgG^+^ memory B cells in *CARD11* GOF patients, as IL-4 has been shown to augment IL-21-induced IgG class-switching [43]. Under the same conditions, IL-4 can inhibit IgA-switching [43]. Accordingly, IgA^+^ memory B cells were preserved in our patient cohort.

An ideal approach to treat CARD11 GOF would be a clinically tested, approved, and efficacious inhibitor of CARD11 itself of downstream MALT1 or NF-κB pathways. However, such inhibitors have not been developed to date. Other than regulating NF-κB signaling, CARD11 is required for TCR-mediated activation of mTORC1, a process that partially depends on glutamine uptake and the amino acid transporter ASCT2 [4, 61]. Convergence of the CARD11 and the PI3K/Akt/mTOR signaling axes probably explains some of the clinical, cellular, and mechanistic similarities between *CARD11* GOF individuals and patients harboring variants that cause overactive PI3K/mTOR signaling, such as the immune dysregulatory disorder Activated PI3K Delta Syndrome (APDS) [62–65]. APDS is caused by *PIK3CD* GOF or *PIK3R1* LOF variants—which encode the p110δ catalytic and p85 regulatory subunits, respectively, of the PI3K heterodimer—resulting in significant and overlapping consequences for lymphocyte biology. *PIK3CD* GOF and *PIK3R1* LOF variants cause multiple aberrations to the B-cell compartment that resemble those observed in *CARD11* GOF. Thus, both CARD11 GOF and PI3K GOF disorders are characterized by defects in B-cell development (increased immature transitional B cells) and maturation including plasmablast differentiation, IgG class-switching, and enhanced B-cell proliferation in response to various stimuli *in vitro* [8, 12, 27, 28]. These shared B-cell intrinsic defects likely contribute to overlapping clinical features of CARD11 GOF and PI3K GOF, such as recurrent sinopulmonary infections, hypogammaglobulinemia, and poor responses to vaccines [65]. Additionally, similarities exist within the T-cell compartment due to CARD11 or PI3K GOF, such as increased CD4^+^FoxP3^+^ cells and enhanced percentages of CD25^−^ cells within this population [27]. Interestingly, increased Akt phosphorylation and reduced expression of FOXO1—the transcription factor which Akt represses—has been observed in settings where pathogenic *CARD11* GOF variants have been introduced into cell lines or mice [7, 66]. Furthermore, sirolimus (rapamycin), a pharmacological inhibitor of mTOR, has been used to treat APDS [67] and has shown varying levels of efficacy in treating CARD11 GOF disease [9, 13, 68]. Thus, some *CARD11* GOF patients showed reductions in lymphadenopathy or splenomegaly (2/4 patients), and an improvement in B-cell lymphocytosis (1/4 patients) in response to sirolimus-mediated mTOR inhibition [9, 13, 68]. More recently, the p110δ-specific inhibitor leniolisib was approved in multiple countries for the treatment of APDS patients over 12 years of age, with clinical trials ongoing for pediatric patients [69, 70]. Given the evidence linking CARD11 and downstream mTOR signaling [4, 26], the lack of a specific inhibitor of CARD11 or NF-κB, and the striking parallels between *CARD11* GOF and PI3K GOF disorders, leniolisib could be explored as a therapeutic option for individuals with immune dysregulation due to activating *CARD11* mutations.

This also raises the possibility of whether targeting the PI3K pathway would be a suitable approach to treat B-cell lymphoma arising from *CARD11* GOF variants [17, 18]. As the pathogenic somatic *CARD11* GOF variants are only present in the malignant lymphoma B cells, there may be deleterious off-target effects of treating this condition with PI3K or mTOR inhibitors. Specifically, there may be excessive inhibition of PI3K/mTOR in WT cells in lymphoma patients, as opposed to regulated attenuation of these pathways in IEIs caused by pathogenic germline variants in *PIK3CD* or *PIK3R1,* where these therapies have been effective [69, 71].

Phenotypic analysis of *CARD11* GOF patients’ T-cell compartments revealed several intriguing features. While there were substantial fluctuations in CD4^+^ and CD8^+^ T-cell subset frequency when comparing individual patients, the frequencies were generally comparable to HD at the cohort level. As noted above, we observed alterations to the CD4^+^FoxP3^+^ population in *CARD11* GOF patients. Interestingly, a similar increase in CD4^+^FoxP3^+^CD25^−^ cells has also been observed in blood from patients and mice harboring PI3K GOF mutations [27] 72], and in SLE patients [73–75]. While the functions of this population remain unclear, there is evidence from mouse studies suggesting CD4^+^FoxP3^+^CD25^−^ cells can later acquire expression of CD25 and perform regulatory functions [76]. Multiple studies have already outlined a role for CARD11 in the regulation of thymic Treg differentiation via NF-κB-dependent and -independent pathways [77–79].

Finally, a unique feature of the T-cell compartment of *CARD11* GOF patients was an expansion of CD45RA^+^CXCR5^+^ T cells, comprising up to ∼15% of the CD4^+^ T-cell pool compared to <2% in HD. In contrast to naïve CD4^+^ T cells, CD45RA^+^CXCR5^+^ CD4^+^ T cells showed enhanced expression of some activation markers (PD-1, CD69), suggestive of heightened basal activation downstream of the TCR due to constitutive CARD11 signaling. Consistent with this, CD45RA^+^CXCR5^+^ CD4^+^ T cells produced higher levels of most tested cytokines than naïve CD4^+^ T cells but substantially lower levels than memory CD4^+^ T cells. It is possible that these CD4^+^ T cells represent pre-Tfh-type cells. Functional analyses of CD4^+^CD45RA^+^CXCR5^+^ T cells in additional patients in future studies will be useful to confirm our findings.

Overall, our study has provided further insights into the role of CARD11 in human B-cell and T-cell biology, and more broadly, its potential interactions with other signaling pathways in the context of lymphocyte development and function. Deep phenotypic profiling of lymphocytes from additional patients harboring GOF variants across the CARD, LATCH, and coiled-coil domains of CARD11 will undoubtedly help to refine and clarify these relationships. Nevertheless, our findings shed light on mechanisms underlying disease pathogenesis due to not only *CARD11* GOF variants but also LOF variants and reveal opportunities to consider targeted therapies such as leniolisib in *CARD11* GOF patients.

## Data Availability

Data included in or related to this manuscript is available on request to the corresponding author.

## Abbreviations:

CARD11: caspase recruitment domain family member 11
mTOR: mechanistic target of rapamycin complex 1
JNK: c-Jun N-terminal kinase
